# The effect of malaria on childhood anemia in a quasi-experimental study of 7,384 twins from 23 Sub-Saharan African countries

**DOI:** 10.3389/fpubh.2022.1009865

**Published:** 2022-12-06

**Authors:** Tim Starck, Peter Dambach, Toussaint Rouamba, Halidou Tinto, Faith Osier, Catherine E. Oldenburg, Maya Adam, Till Bärnighausen, Thomas Jaenisch, Caroline A. Bulstra

**Affiliations:** ^1^Heidelberg Institute of Global Health, Heidelberg University Hospital, Heidelberg, Germany; ^2^Clinical Research Unit of Nanoro, Institut de Recherche en Sciences de la Santé, Nanoro, Burkina Faso; ^3^Centre for Infectious Diseases, Parasitology, Heidelberg University Hospital, Heidelberg, Germany; ^4^KEMRI-Wellcome Trust Research Programme, Centre for Geographic Medicine Research-Coast, Kilifi, Kenya; ^5^Francis I Proctor Foundation, University of California, San Francisco, San Francisco, CA, United States; ^6^Department of Ophthalmology, University of California, San Francisco, San Francisco, CA, United States; ^7^Department of Epidemiology and Biostatistics, University of California, San Francisco, San Francisco, CA, United States; ^8^Deptartment of Pediatrics, Stanford School of Medicine, Stanford, CA, United States; ^9^Department of Global Health and Population, Harvard T.H. Chan School of Public Health, Boston, MA, United States; ^10^Africa Health Research Institute (AHRI), KwaZulu-Natal, South Africa; ^11^Center for Global Health, Colorado School of Public Health, Aurora, CO, United States; ^12^Department of Epidemiology, Colorado School of Public Health, Aurora, CO, United States; ^13^Department of Public Health, Erasmus MC, University Medical Center Rotterdam, Rotterdam, Netherlands

**Keywords:** twins, multiples, fixed-effect, Sub-Sahara Africa, hemoglobin, DHS, anemia-etiology, malaria

## Abstract

**Background:**

Young children in Sub-Saharan Africa (SSA), particularly those from resource-limited settings, are heavily burdened by anemia and malaria. While malaria infected children frequently become anemic (hemoglobin < 110 g/L), anemia is a strongly multifactorial disease with many other risk factors than malaria. Due to the complex and often overlapping contributors to anemia, it remains challenging to isolate the true impact of malaria on population level hemoglobin concentrations.

**Methods:**

We quantified the malaria-induced effect on hemoglobin levels in children under 5 years of age, leveraging data from 7,384 twins and other multiples, aged 6 to 59 months, from 57 nationally representative Demographic and Health Surveys (DHSs) from 23 SSA countries from 2006 to 2019. The quasi-experimental twin fixed-effect design let us minimize the impact of potential confounders that do not vary between twins.

**Results:**

Our analyses of twins revealed a malaria-induced hemoglobin decrease in infected twins of 9 g/L (95% CI -10; -7, *p*<0.001). The relative risk of severe anemia was higher (RR = 3.01, 95% CI 1.79; 5.1, *p*<0.001) among malaria positive children, compared to malaria negative children. Conversely, malaria positive children are only half as likely to be non-anemic (RR = 0.51, 95% CI 0.43; 0.61, *p*<0.001).

**Conclusion:**

Even after rigorous control for confounding through a twin fixed-effects study design, malaria substantially decreased hemoglobin levels among SSA twins, rendering them much more susceptible to severe anemia. This effect reflects the population-level effect of malaria on anemia.

## Introduction

Young children from low- and middle-income countries frequently suffer from a wide range of health burdens specific to their under-resourced settings. In Sub-Saharan Africa (SSA) these often include anemia of multifactorial origin and endemic infectious diseases such as malaria, where recent estimates have suggested a prevalence of up to 60% (Supplementary material S1) (1–4). Childhood anemia (WHO definition: hemoglobin < 110 g/L) has complex etiologies, including malaria, socioeconomic factors, nutritional deficits like iron deficiency, genetic disorders such as sickle cell disease, and other infectious diseases including helminths, intestinal and respiratory diseases (1, 2, 5, 6). Anemia impairs the cognitive and physical development of young children, subsequently reducing educational achievements, labor market opportunities and, ultimately, perpetuating cycles of poverty by limiting the potential of young generations to flourish (7–10). Prieto-Patron et al. (11) estimated the total average cost of iron deficiency anemia in Cote d'Ivoire alone to exceed 890 million USD (2.5% of GDP), and 214 700 disability-adjusted life years (11). Assuming that iron deficiency anemia constitutes only one quarter to one half of the total anemia burden in SSA, the projected total costs of anemia (all etiologies) are devastatingly high (12). Furthermore, according to a recent analysis malaria might account for up to a third of iron-deficiency anemia cases in endemic countries (13).

Malaria infections frequently lead to anemia, acutely, through erythrocyte hemolysis, and in chronic and recurring forms through persisting bone-marrow depression and inflammation (14–19). Malaria is caused by the *Plasmodium* parasite genus whose species differ in both geographic distribution and severity of the resulting disease (20, 21). Globally, more than 95% of annual malaria cases occur in SSA, partly because it is almost exclusively affected by the particularly aggressive *Plasmodium falciparum* (22–24). In 2020 *P. falciparum* caused over 227 million cases of malaria in SSA, with 602 000 fatal outcomes out of which 80% were children younger than 5 years old (24). Despite the clear links between malaria and anemia, the true impact of malaria on population hemoglobin levels is obscured by the complex and often overlapping contributors to anemia.

**Figure 1:**
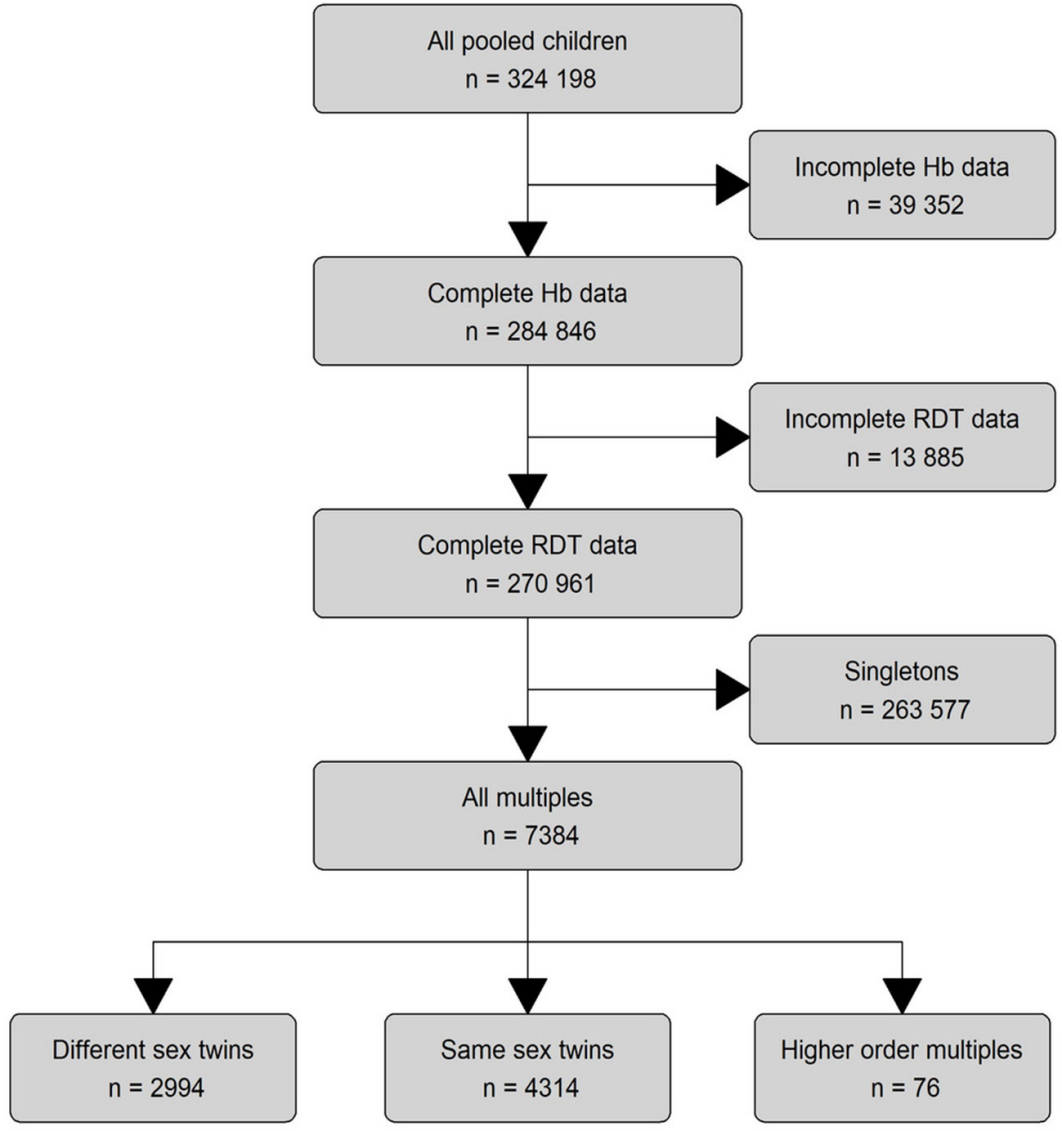
Eligibility assessment and data processing. We pooled 324,198 children from all available DHS surveys (57 surveys from 23 countries) that contained hemoglobin and malaria outcomes. We analyzed the data on three different levels: The main descriptive analysis was performed on all children with complete RDT data (n = 270,961). The main regression model was run on all multiples (n = 7,384), while the subset analyses were performed on the lowest level for different-sex twins alone (n = 4,314) and same-sex and higher level multiples combined (n = 3,070). Blind ended boxes show cases that did not meet subset criteria and were not considered in any analysis. Boxes with continued lines show cases used for analysis.

To bridge this knowledge gap and isolate the impact of malaria on hemoglobin, we pooled nationally representative survey data from 23 SSA countries. Mining these datasets for pairs of twins and higher order multiples (triplets and quadruplets; for ease of reading henceforth also referred to as “twins”, Figure 1) offered the opportunity to account for factors that are only shared between twins and are otherwise nearly impossible to measure in large trials such as *in-utero*, perinatal and early childhood exposures (25, 26). To achieve this level of control, we adapted an econometric statistical method for our main analysis, fixed effect regression, to rigorously control for most of the major risk factors of anemia that are identical between twins, including temporal and regional constants, mother-dependent variables, and socioeconomic status (25–29).

Our study aims to isolate the exact malaria-induced change in hemoglobin levels in endemic settings of SSA. This is currently of particular relevance as recent promising malaria-vaccine trials and the consecutive recommendation of the WHO to vaccine young children give hope of an imminent improvement of the malaria burden to endemic countries (24, 30, 31). Our study will shed light on the potential gains against anemia secondary to an improved or successful malaria elimination process.

## Methods

### Data and variables

**Figure 2:**
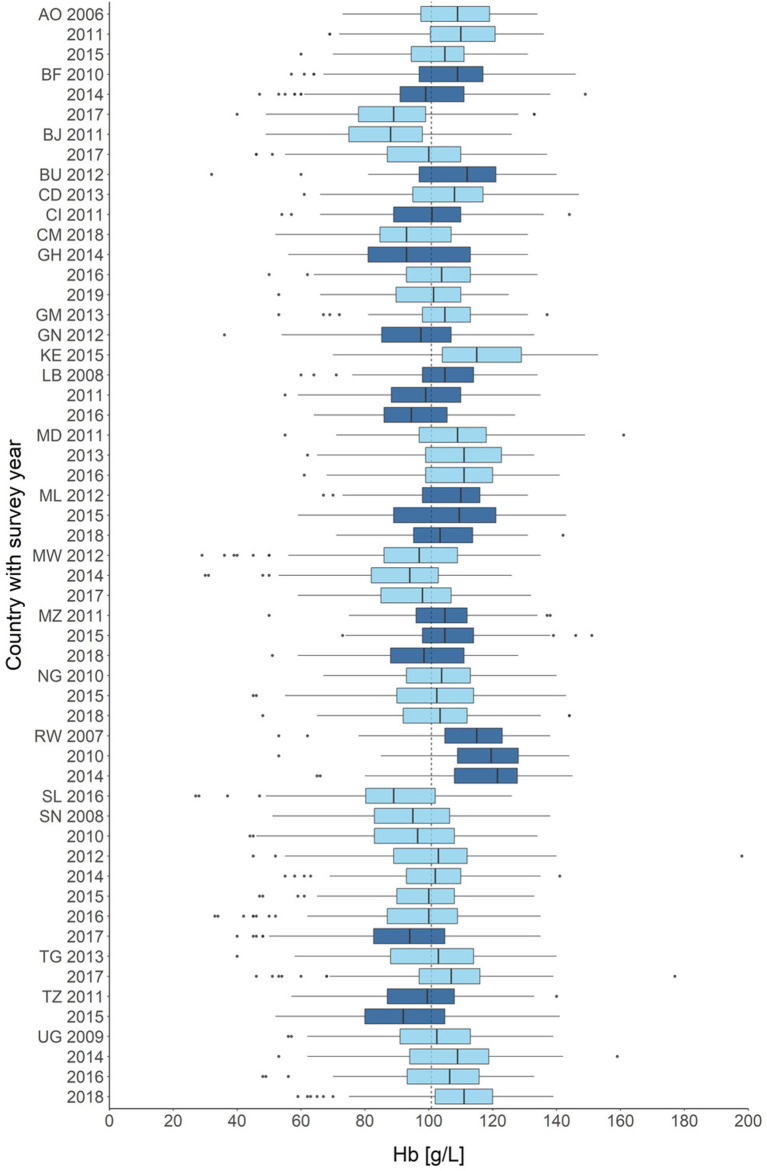
Distribution of hemoglobin across all children in all included surveys (n = 270,961). Countries are color-coded, surveys with years are given on the y-axis. Hemoglobin concentrations are given in g/L on the x-axis. AO, Angola; BF, Burkina Faso; BJ, Benin; BU, Burundi; CD, Côte d'Ivoire; CM, Cameroon; GH, Ghana; GM, Gambia; GN, Guinea; KE, Kenya; LB, Liberia; MD, Madagascar; ML, Mali; MW, Malawi; MZ, Mozambique; NG, Nigeria; RW, Rwanda; SL, Sierra Leone; SN, Senegal; TG, Togo; TZ, Tanzania; UG, Uganda. Survey year is set when the survey was begun, some surveys crossed over into the following years.

We used nationally representative survey data which are available through USAID's Demographic and Health Surveys (DHSs) and Malaria Indicator Surveys (MISs) (DHS Program, RRID:SCR_000905). During the surveys, trained field workers conducted extensive interviews, collected biomarkers, and gathered anthropometric data from a representative cluster-based sample of households in the respective survey country (Figure 2). All households and participants receive new identifiers for each survey, thus the pooled data is strictly cross-sectional, as it is impossible to trace participants across surveys. All data and details on the data generation process are freely available from the DHS website (32).

All surveys from the SSA region that contained hemoglobin measurements and rapid diagnostic tests (RDT)-based malaria measurements were eligible for analysis. We downloaded the survey data using the *rdhs (v0.7.1)* R package and extracted the variabes sex, hemoglobin and RDT result for analysis. Furthermore, we extracted the survey identifier, the household identifier, the date of birth and the mother identifier to identify the twin pairs. The datasets were restricted to children with complete RDT and hemoglobin measurements.

We limited the data to children aged 6–59 months as younger children typically benefit from pregnancy related IgG-transfers, i.e., they are still protected by the mother's immune system, while older children will have had significantly more exposure to malaria and begin to develop partial immunity of their own (26, 33). We further regionally restricted the data to Sub-Saharan Africa because of its high prevalence of malaria and almost exclusive endemicity of *P. falciparum* (22, 23). We used blood hemoglobin levels as a proxy for anemia in line with the WHO definition of anemia for children (hemoglobin ≥110 g/L healthy; 109–100 g/L mild anemia; 99–70 g/L moderate anemia; < 70 g/L severe anemia) (5). Hemoglobin levels were measured using HemoCue test systems (32).

We used the malaria test results from established RDT systems to identify the children with parasitemia, understood as a binary indicator for parasite presence. Unfortunately, there was no quantitative measurement of parasitemia (i.e., parasite density) available in the given data. RDTs are frequently used in remote clinical settings and surveys because they work well-with minimal laboratory equipment while providing solid and immediate results on site. The tests detect *plasmodium*-specific antigens in a lateral flow system to diagnose malaria (34, 35).

Unfortunately, the data did not contain reliable information on potentially confounding causes of anemia such as iron status, coinfections or genetic disorders.

### Data preparation

The flowchart illustrates the process of data preparation and eligibility assessment (Figure 1). The eligibility criteria of SSA-region, hemoglobin and RDT-results were met by 57 surveys from 23 countries, spanning a time frame from 2006 to 2019 (Figure 2).

Since there was no predefined twin-identifier in the given data, we manually constructed a twin-identifier variable from the country code, survey year, cluster identifier, household identifier, mother identifier and date of birth (century month code) of the child, thus ensuring that only twins and higher order multiples shared a common indicator. The resulting main dataset contained 7,384 monozygotic and dizygotic twins with complete data and was used in the analysis of the main model.

With the given data it is not possible to exclusively identify monozygotic twins. Instead, we approached a selection of monozygotic twins by creating a subset excluding twin pairs with differing sex and all multiples (same-sex and different-sex alike), to maximize the probability of genetic similarity (36). This subset of same-sex twins included 4,314 individuals out of which 1,320 (30.6%) will likely be monozygotic. We provide a more in depth explanation of this approach in the Supplementary material S2.

### Statistical analysis

We first described the overall patterns in the population structure of all children and of the twin subsets to enable easier comparison between the groups and assess the representativeness of the twins.

We further added a visual analysis of the hemoglobin distributions among all children for each included survey and created prevalence maps for anemia and malaria for the most recent survey in every country (Supplementary material S1). We used only the twins and sex-subsets for the inferential analysis, not the pooled data of all children. For each of the three regression datasets (*main, same-sex, different-sex*) we constructed a linear twin-fixed effect regression model. Each of the models estimated the expected difference in hemoglobin of twins with parasitemia compared to those without, with a separate intercept for each pair of twins. In more descriptive terms, the model considers a twin pair as a single entity, where the individual siblings within a twin pair represent two simultaneous measurements of the same entity in two different exposure-states (25, 28). The quasi-experimental objective was to analyse the parasitemia-associated hemoglobin change in two counter-factual realities of one child (as represented through a twin pair). The twin pairs that did not differ in parasitemia status could not be used to estimate the hemoglobin change, however they provided power to the overall analysis. This approach is increasingly popular in epidemiologic research and allowed us to minimize the impact of potential confounders that do not vary between twins, including most relevant risk factors of anemia (27, 29). The formula for the main model is provided with the Supplementary material S3.

We adjusted our *main* and *different-sex* models for sex and the twin fixed-effect, the *same-sex* model for the twin fixed-effect only. All variables that did not vary between twins, such as country, age, test method, etc. were perfectly colinear with the twin-fixed effect and were therefore excluded from the statistical model (28). We further added subset analyses for each individual country and stratified by age group to account modulating effects of these factors (Supplementary material S4).

The quasi-poisson models are four separate extensions of the main model structure that allowed us to estimate the malaria effect on the relative risk of mild or worse (< 110 g/L), moderate or worse (< 90 g/L) and severe anemia (< 70 g/L). These analyses illustrate in a more tangible way the consequences that are implied in the malaria-induced hemoglobin reductions. All analyses were performed using R version 4.0.2. We extracted, cleaned and analyzed the data with the help of the following r packages (rdhs 0.6.3; plm 2.2.4, fixest 0.8.3).

## Results

An overview of the demographic composition of the datasets used for analysis is provided in Table 1. Demographic summaries of each survey are provided in the Supplementary materials S5, S6. Across all surveys, most children (62.9%) suffered from at least mild anemia (Hb < 110 g/L). The prevalence of anemia was higher in *plasmodium* positive children than in negative ones (82.4 vs. 56.2%) (Supplementary material S7). The average hemoglobin across the included surveys decreased marginally but statistically significant over time (2006–2010: 106 g/L, 2015–2019: 103 g/L; unpaired *T*-test: *p*<0.001). The pooled prevalence of *plasmodium* parasitemia also remained largely constant between survey years (2006–2010: 18.4%, 2015–2019: 18.8%; two proportions Z-test: *p* = 0.002). The distribution of hemoglobin in each survey is shown in Figure 2. In total 547 (14.89%) of the total 3,679 twin pairs had disconcordant parasitemia status, which was leveraged for the fixed-effect model. Finally, there was no statistically significant difference in the hemoglobin distributions of same-sex and different-sex twins in a two sample unpaired *T*-test (95% CI -0.7; 0.9, *p* = 0.808).

**Table 1:** Demographic and health characteristics of the study populations, separately for each level of analysis.

	**All children**	**All twins**	**Same-sex twins**	**Mixed-sex twins**
**Total**	270,961 (100)	7,384 (100)	4,314 (100)	3,070 (100)
**Sex (%)**				
Male	136,710 (50.5)	3,594 (48.7)	2,066 (47.9)	1,528 (49.8)
Female	134,251 (49.5)	3,790 (51.3)	2,248 (52.1)	1,542 (50.2)
**Age**				
0–11 months	29,073 (10.7)	883 (12)	533 (12.4)	350 (11.4)
12–23 months	59,577 (22)	1,678 (22.7)	993 (23)	685 (22.3)
24–35 months	59,593 (22)	1,597 (21.6)	936 (21.7)	661 (21.5)
36–47 months	61,685 (22.8)	1,587 (21.5)	884 (20.5)	703 (22.9)
48–59 months	61,033 (22.5)	1,639 (22.2)	968 (22.4)	671 (21.9)
**Multiples (%)**				
Twins	7,308 (2.7)	7,308 (99)	4,314 (100)	2,994 (97.5)
Triplets	72 (0)	72 (1)	-	72 (2.3)
Quadruplets	4 (0)	4 (0.1)	-	4 (0.1)
**Malaria (%)**				
Negative	201,524 (74.4)	5,445 (73.7)	3,233 (74.9)	2,212 (72.1)
Positive	69,437 (25.6)	1,939 (26.3)	1,081 (25.1)	858 (27.9)
**Anemia (%)**				
Hb ^a^, ^b^	103 (17)	101 (18)	101 (18)	101 (18)
No anemia	100,542 (37.1)	2,453 (33.2)	1,435 (33.3)	1,018 (33.2)
Mild anemia	116,268 (42.9)	3,094 (41.9)	1,803 (41.8)	1,291 (42.1)
Moderate anemia	44,763 (16.5)	1,500 (20.3)	881 (20.4)	619 (20.2)
Severe anemia	9,388 (3.5)	337 (4.6)	195 (4.5)	142 (4.6)
**Malaria positives by anemia (%)**				
No anemia	12,242 (12.2)	362 (14.8)	196 (13.7)	166 (16.3)
Mild anemia	30,371 (26.1)	821 (26.5)	454 (25.2)	367 (28.4)
Moderate anemia	20,915 (46.7)	583 (38.9)	337 (38.3)	246 (39.7)
Severe anemia	5,909 (62.9)	173 (51.3)	94 (48.2)	79 (55.6)
**Anemia by malaria status (%)**				
No malaria	113,224 (56.2)	3,354 (61.6)	1,994 (61.7)	1,360 (61.4)
Malaria	57 195 (82.4)	1,577 (81.3)	885 (81.9)	692 (80.7)

Column one represents is based on data from all children from all included surveys. Column two is based on data from all twins in the main regression model. Columns three and four are based on the subset regression analyses for same-sex and different-sex twin pairs, respectively. Based on the demographics, twins are representative for all children in the data. Percentages might not add up to 100 due to rounding to the first decimal.

^a^Given as mean (standard deviation);

^b^unit [g/L]; Anemia severity categories are based on hemoglobin levels in g/L. No anemia (≥110 g/L), mild anemia (90–109 g/L), moderate anemia (70–99 g/L), severe anemia (< 70 g/L).

**Figure 3:**
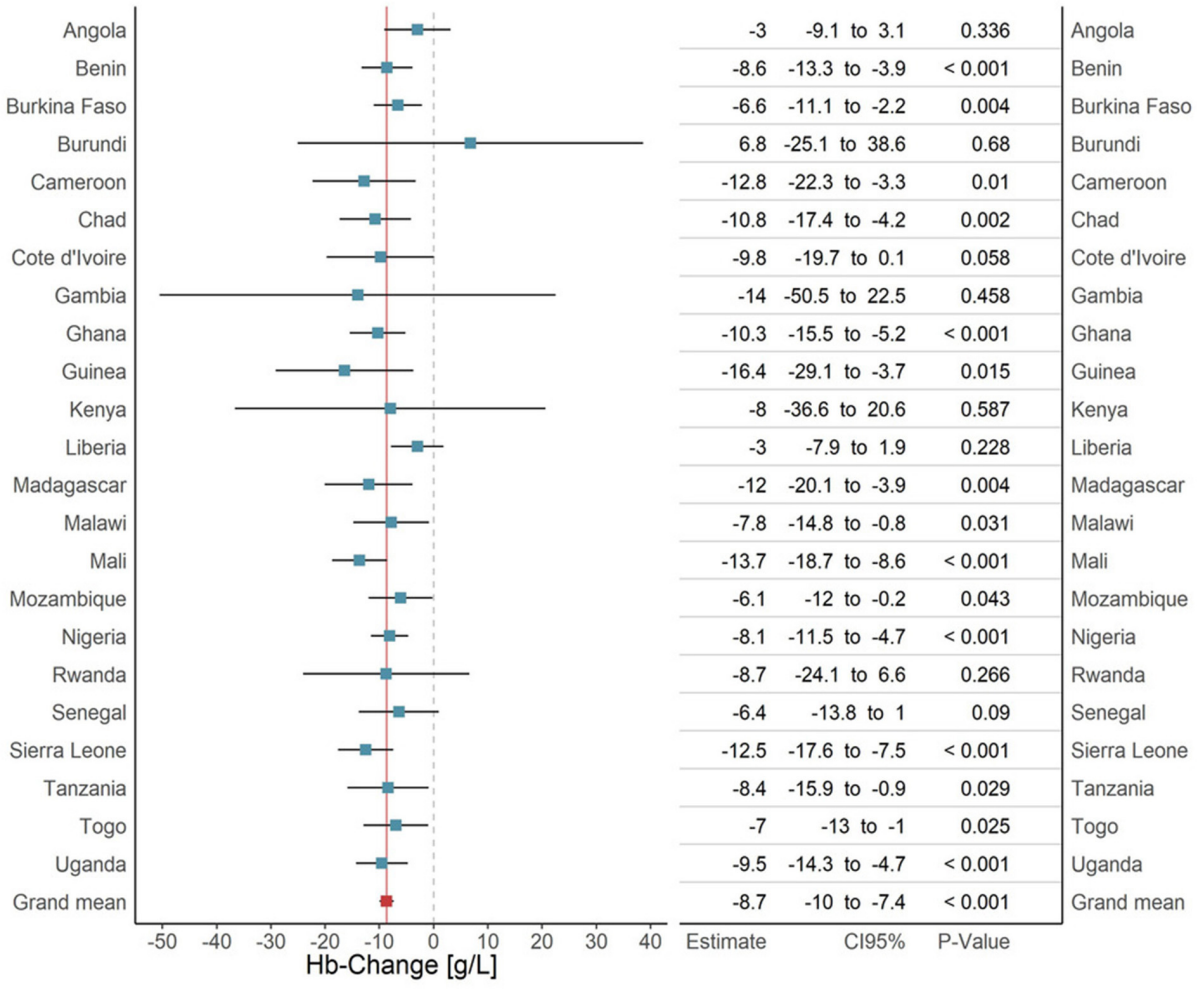
Analysis of the malaria effect on hemoglobin, overall effect and country specific effects. Each bar represents the result of a subset-analysis that used all surveys of a given country (blue). The errorbars represent the corresponding 95% confidence intervals. The bottom row (red) shows the outcome of the main pooled analysis across all twins from all surveys in all countries. The continuous dark red vertical line is the extension of the main pooled analysis result. The dashed line is the line of no effect. The table to the right adds the exact regression results for every country with 95% confidence intervals and p-values.

The *main* model, adjusted for sex and the twin fixed effect, showed a 9 g/L (95% CI -10; -7, *p*<0.001) hemoglobin decrease in children with positive RDT compared to their RDT-negative twins. Female sex increased hemoglobin by 2 g/L (95% CI 1; 3, *p*<0.001). The main model outcome and the outcomes for each separate country analysis are illustrated in Figure 3, the age subset analysis is appended to the Supplementary materials S4, S8.

We adjusted the second, the *same-sex* model, for the twin fixed effect only and found a malaria-induced hemoglobin decrease of 9 g/L (95% CI -11; -7, *p*<0.001). Based on the Weinberg rule we estimated that a third of the same-sex data set are monozygotic twins (Supplementary material S2). The monozygotic twins thus weigh more heavily in the *same-sex* model than in the *main* model data where the monozygotic twins likely represent around one fifth.

The third, the *different-sex* model, was adjusted for the twin fixed effect and sex and included only those twins who were definitely dizygotic. We found a 8 g/L (95% CI −10; −6, *p*<0.001) hemoglobin reduction induced by *plasmodium* presence among the different-sex twins. The effect of parasitemia in the *different-sex* model is thus marginally smaller than the effect of the *main* and *same-sex* model (Supplementary material S9).

Based on the main model we also conducted a quasi-poisson regression with anemia severity as binary outcomes. In this model series *plasmodium* positive children where at higher relative risk of mild anemia or worse (Hb < 110 g/L), (RR = 1.28, 95% CI 1.2; 1.36, *p*<0.001), moderate anemia or worse (Hb < 90 g/L), (RR = 1.76, 95% CI 1.49; 2.08, *p*<0.001) and severe anemia (Hb < 70 g/L), (RR = 3.01, 95% CI 1.79; 5.1, *p*<0.001). At the same time, their “risk” of being healthy (Hb≥110) was reduced (RR = 0.51, 95% CI 0.43; 0.61, *p*<0.001). The outcomes of the quasi-poisson regression are illustrated in Supplementary material S10.

## Discussion

Our models consistently demonstrated a malaria-induced reduction in hemoglobin when comparing *plasmodium*-infected children to their healthy twin counterparts and confirmed that malaria substantially impacts population-level hemoglobin. *Plasmodium* presence was associated with a 9 g/L hemoglobin decrease among all twins and among the same sex twins. Among the different-sex twins the parasitemia-associated hemoglobin decrease was marginally smaller. Since the anemia severity groups are distinguished by steps of 20 g/L hemoglobin, the effect sizes are large enough to be clinically relevant, particularly if considering that our calculations are based on cross-sectional data. This is also illustrated in the poisson regression, where children with parasitemia are only about half as likely to be non-anemic and three times more likely to be severely anemic than their *plasmodium*-negative peers. The slightly larger effect of the *same-sex* model in comparison to the *different-sex* model might point toward a relevant genetic predisposition, e.g., sickle cell anemia, G6PD-deficiency or thalaessaemias (13, 37). However, the hemoglobin difference between the groups is neither statistically significant nor clinically relevant.

To our knowledge, this is the first large scale twin study on the impact of malaria on anemia. It complements an extensive body of research on the clinical impact of malaria on individual, and, to a lesser extent, on population hemoglobin. Previous research has described the population impact in a wide range from -13 g/L (WHO, 2019 malaria report), to a marginal -1 g/L among adults in a regional setting in south-east Asia (38, 39). *In vivo* studies have described the course of anemia during a *plasmodium* infection in adults with a sharp initial drop of -15 g/L hemoglobin followed by a rapid recovery to a plateau of -5 g/L (40). Korenromp et al. found successful malaria control strategies to improve population hemoglobin in children by 7 g/L (41). Our point estimate is located at the center of the range described in the literature and we confidently attribute this to the strict control for confounding in our modeling approach. The sex-associated hemoglobin difference is not backed well by current literature. Normally, we would expect to see a difference at the onset of puberty, not in children younger than five. However, sex dependent differences in early childhood have been recently described by Fulgoni et al. and are in line with our previous findings in Burkina Faso (42, 43). The high prevalence of anemia among malaria-negative children is within the expected expected range given the regional circumstances and has been previously described in other large studies (4, 44, 45).

Our study benefits from several strong innovations. It draws data from a large, representative sample of children in SSA, the region where the greatest gains against malaria have been made and yet much remains to be done. Additionally, from this representative sample we identified a very large number of twins as a basis for our fixed effect statistical model, controlling for most relevant confounding that is shared between twins (25, 28).

Our study has several limitations. Firstly, the nature of cross-sectional data is vulnerable to certain types of bias. This includes survival bias, where children with severe anemia are likely treated in clinics to receive intensified medical care and therefore cannot be included in the surveys (20). We expect survival bias to modify our results toward a more conservative estimation. Furthermore, the manual construction of the twin identifier could possibly introduce classification bias, where a child with an absent or deceased twin sibling is not correctly identified as a twin.

Secondly, we decided to use RDT result as our indicator for malaria status, rather than microscopy although microscopy is considered the gold standard. We made this choice because the RDTs most effectively reflect the diagnostic situation in SSA. RDTs are used in most DHS surveys, are a staple in clinical settings of rural Africa where microscopy is frequently not feasible and have very similar sensitivity and specificity (46, 47). The downside of RDTs is that they are susceptible to false positives, particularly in the presence of rheumatoid factor and in latent malaria infections (48–50). Some RDTs have been shown to remain positive in children for up to 30 days even after an infection is under immune system or medical control (51). Since the included survey time span ranges from 2006–2019, the quality of deployed RDTs will likely have improved over the years (52). The reliance on RDTs that developed over time, the different test models and target-antigens used, pose a risk of systematic measurement erros that can't be fully addressed through the study design as the error rates might vary between surveys.

Thirdly, we were not able to exclusively identify monozygotic twins within the given data set. This is important because the genetic relationship of dizygotic twins is roughly that of regular siblings, therefore our main model does not control for genetic differences that might vary between twins and still cause anemia, e.g., sickle-cell anemia trait. However, the twin fixed-effect approach is superior to a mother- or household-fixed-effect approach because it controls for all temporal and regional covariates that are associated with the twin birth, including *in-utero* and perinatal exposures, the mother's education, location of birth, age of the child, early childhood exposures etc (26). Similarly, the models lack explicit control for coinfections and malnutrition which were not covered in the data but might differ between twins. However, since these potential confounders are likely not systematically linked to the malaria status, their impact on the final results should be limited. Nevertheless, future surveys will hopefully address these gaps in the data to enable a clearer picture on anemia in the study populations.

The burden of anemia and malaria to SSA countries is substantial (53). However, the combined risks of malaria and anemia put affected children in a double-jeopardy situation where those that survive the acute infection frequently suffer anemia with corresponding detriments to development and increased infectious disease susceptibility (54, 55). In the long run, this close interaction can perpetuate cycles of poverty where anemia diminishes a child's chances at future upward social mobility and their future offspring will therefore be exposed to poverty, malaria, and anemia again. Hence, successfully combating malaria reduces the burden of anemia and proffers critical secondary benefits for the health and development of children and, ultimately, the economic stability of their countries. These benefits do not seem to be as far out of reach anymore as a first malaria vaccine gives hope of improved success in decreasing the burden of malaria and secondary to that, the burden of anemia (31).

In conclusion, we were able to demonstrate the substantial, malaria-induced reduction of population hemoglobin in one of the largest studies of SSA twins to date, while rigorously controlling for almost all confounders that are relevant to anemia and malaria. Since malaria's impact on hemoglobin reflects up to the population level it can be reasonably assumed that malaria delays development of individuals, regions, and entire countries. The work on eradicating malaria remains valuable, particularly those efforts that target the high risk group of young, anemic children. These programs should be extended rather than neglected, despite the current difficulties in the global political and health landscape.

## Data availability statement

Publicly available datasets were analyzed in this study. This data can be found at: https://dhsprogram.com/ (RRID:SCR_000905).

## Author contributions

TS and TB designed the study. TS performed the data analysis and drafted the manuscript with support of CB. All co-authors contributed significantly to the revision of the manuscript and provided scientific guidance. All authors contributed to the article and approved the submitted version.

## Funding

For the publication fee we acknowledge financial support by Deutsche Forschungsgemeinschaft within the funding programme Open Access Publikationskosten as well as by Heidelberg University and the Heidelberg Institute of Global Health.

## Conflict of interest

The authors declare that the research was conducted in the absence of any commercial or financial relationships that could be construed as a potential conflict of interest.

## Publisher's note

All claims expressed in this article are solely those of the authors and do not necessarily represent those of their affiliated organizations, or those of the publisher, the editors and the reviewers. Any product that may be evaluated in this article, or claim that may be made by its manufacturer, is not guaranteed or endorsed by the publisher.
