# Supplementary material for: The effect of malaria on childhood anemia in a quasi-experimental study of 7,384 twins from 23 Sub-Saharan African countries
Source: Front Public Health. 2022 Dec 6;10:1009865. doi: 10.3389/fpubh.2022.1009865 (PMC9766366; doi:10.3389/fpubh.2022.1009865)
Supplement: Supplementary file 1 [file Data_Sheet_1.docx]

**Online-Only Supplement**

Online only supplement including **S1 – S10.**

**S1. Map of malaria and anemia prevalence. A** Most recent ^a^ malaria prevalence based on the pooled survey data for all 23 countries included in the study. **B** Most recent ^a^ prevalence of moderate to severe anemia (Hb < 90 g/L) based on the pooled survey data for all 23 countries included in the study.


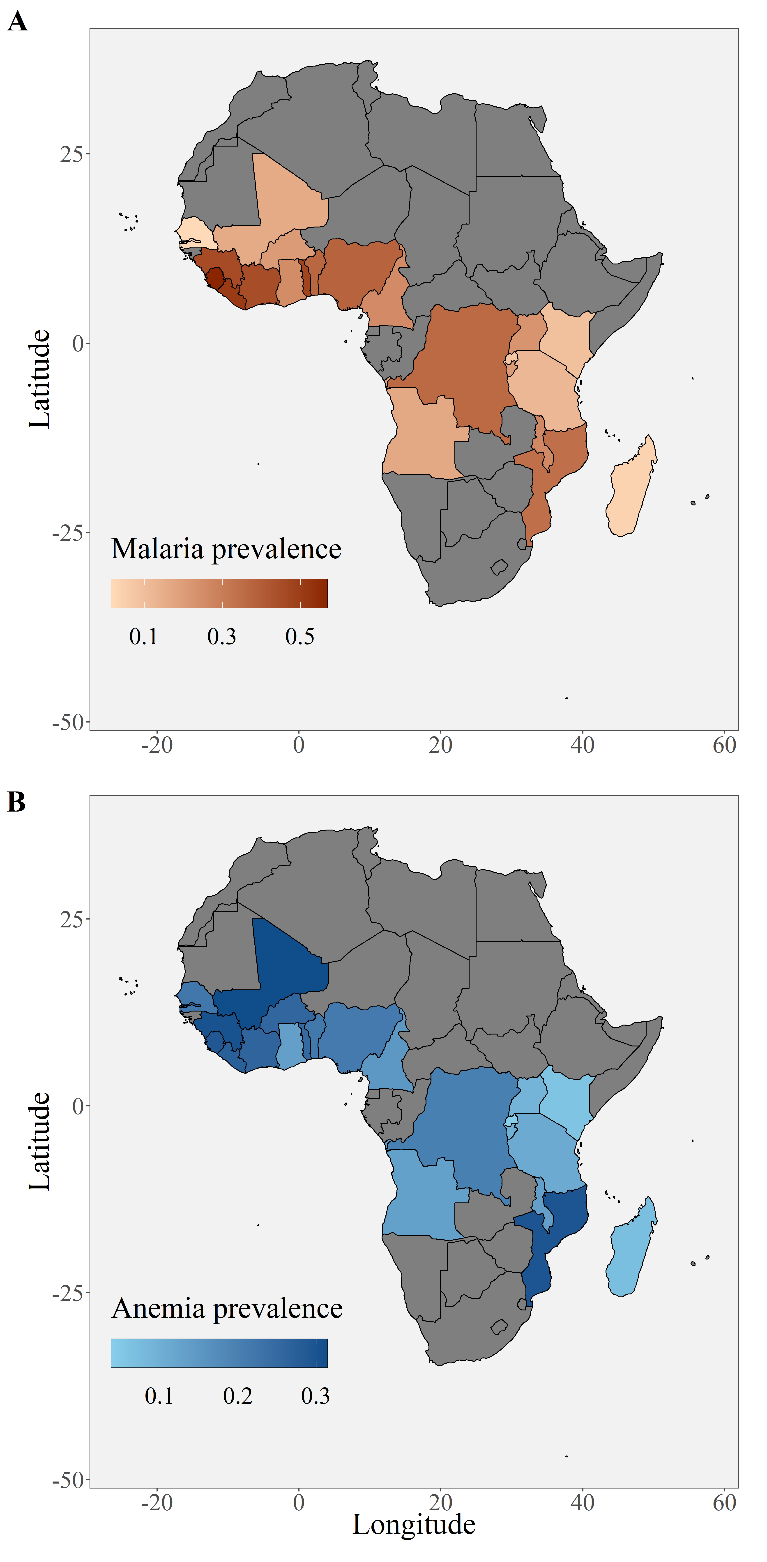


*^a^*Most recent surveys: Angola 2016, Benin 2018, Burkina Faso 2018, Burundi 2013, Cameroon 2019, Democratic Republic of Congo 2014, Côte d’Ivoire 2012, Gambia 2013, Ghana 2019, Guinea 2012, Kenya 2015, Liberia 2016, Madagascar 2016, Malawi 2017, Mali 2018, Mozambique 2018, Nigeria 2018, Rwanda 2015, Senegal 2017, Sierra Leona 2016, Tanzania 2016, Togo 2017, Uganda 2019)

**S2. The Weinberg rule, estimation of monozygotic frequency.**

The idea of subsetting all twins for only same-sex pairs is based on the Weinberg rule which dictates that a pair of dizygotic twins is equally likely to be of the same sex as of differing sex. ^1^ Monozygotic twins on the other hand are necessarily always of the same sex. Same-sex multiples of higher order are also excluded from this subset because they are typically at least dizygotic.

The estimated number of monozygotic twins in the data therefore employs the following formula:

$$\hat{n}_{MZ}=n_{T}-2 \times n_{DS}$$

Where $n_{T}$ (7308) is the total number of twins (excl. multiples)
$n_{DS}$ (2994) is the number of different-sex
$\hat{n}_{MZ}$ (1320) is the expected number of monozygotic twins (30.6%) among all same sex twins.

Subsetting the data for only same-sex twins allowed us to substantially increase the proportion of monozygotic twins in the analysis, increasing genetic similarity and reducing the impact of genetic similarity. In the main analysis monozygotic twins represented 18.1% of cases, whereas in the same-sex analysis 30.6% of cases are expected to be monozygotic.

**S3. Main regression model formula**

Our *main* model employed the following formula:

*Y_it_= α_t_ + βM_it_ + γX_it_ + μ_t_ + ε_it_*

Where *Y_it_* is the Hb concentration of twin *i* within twin pair *t*.

*α_t_* is the intercept

*β* is the association between Hb concentration and malaria status.

*M_it_* is child i’s malaria test result.

*γ* is the association between sex and Hb concentration.

*X_it_* is the child’s sex

*μ_t_* is the twin fixed effect, representing unmeasured, twin-invariant confounding

*ɛ_it_* is the residual unexplained individual-level variation.

**S4**. **Age Group subset analysis**

We ran an additional subset analysis based on the age groups 6 – 12 months, 13 – 24 months, 25 – 36 months, 37 – 48 months and 49 – 59 months. This allowed us to account for possible age modulated effects that couldn’t be assessed in the main model structure, as age is perfectly colinear within a pair of twins but might still modulate the effect malaria has on hemoglobin.

We found a clear age modulation of the malaria effect on hemoglobin. The impact of age on the malaria effect on hemoglobin decreased continuously from the youngest (6 -11 months, -11 g/L, CI95% -1.5 to -7, P < 0.001) to the one year olds (12 – 24, -9 g/L, CI95% -1.2 to -6, P < 0.001) to the two year olds (25 – 36 months, -7 g/L, CI95% -10 to -4, P < 0.001) (**eFigure2)**. However, in the fourth age group (37 – 48 months, -10 g/L, CI95% -13 to -7, P < 0.001) the age modulated effect increased again, only to decrease among the oldest children (49 -59 months, -8 g/L, CI95% -11 to -6, P < 0.001). As shown in of the main manuscript **Table 1** the group sizes are balanced and this effect is likely not attributable to statistical artifacts. However, these results should still be interpreted with caution due to the wide and overlapping confidence intervals of the subset analyses.

The pattern of age modulation from the subset analysis is unexpected and hard to attribute to statistical artifacts or bias since the age groups are well balanced across countries and malaria prevalence. We therefore assume that the observed pattern mirrors the cumulative effect of consecutive malaria episodes among the first years of life. Multiple heterologous consecutive malaria infections can cause progressively worse anemia if the previous infection has not healed completely or persists on a subclinical level.^2-4^ As this effect can only occur with time after several recurring episodes and before immunity builds, it is unlikely to impact the youngest or oldest age groups but would leave the middle age groups vulnerable. However this is hard to prove in our data as historic data of malaria episodes is lacking and this aspect warrants further research and investigation.

**S5. Characteristics of all children in the pooled surveys.** Describes demographic characteristics for children within each included survey. ^a^Prevalence of moderate to severe anemia in the study population (Hb < 90 g/L). ^b^Prevalence of moderate to severe anemia (< 90 g/L) among malaria positive children. ^c^Democratic Republic of Congo.

| **Survey** | | **Children** | **Female** | **Age (months)** | **Malaria Positive** | **Hb [g/L]** | **Pv Anemia^a^** | **Pv Anemia^b^** |
| --- | --- | --- | --- | --- | --- | --- | --- | --- |
| **Country** | **Year** | **N** | **N (%)** | **Mean (SD)** | **N (%)** | **Mean (SD)** | **N (%)** | **N (%)** |
| **Angola** | 2007 | 2300 | 1132 (49.2) | 32.2 (15.6) | 539 (23.4) | 106 (15) | 264 (11.5) | 122 (22.6) |
|  | 2011 | 3413 | 1760 (51.6) | 32.1 (16) | 424 (12.4) | 109 (15) | 306 (9) | 116 (27.4) |
|  | 2016 | 6814 | 3374 (49.5) | 32.2 (15.4) | 1126 (16.5) | 105 (16) | 883 (13) | 384 (34.1) |
| **Benin** | 2012 | 3747 | 1823 (48.7) | 33.3 (15.4) | 1015 (27.1) | 105 (17) | 587 (15.7) | 286 (28.2) |
|  | 2018 | 6247 | 3087 (49.4) | 32.3 (15.9) | 2309 (37) | 101 (15) | 1351 (21.6) | 870 (37.7) |
| **Burkina Faso** | 2010 | 5948 | 2892 (48.6) | 32.2 (15.3) | 4499 (75.6) | 90 (17) | 2773 (46.6) | 2174 (48.3) |
|  | 2014 | 6150 | 3020 (49.1) | 32.5 (15.4) | 3962 (64.4) | 90 (17) | 2925 (47.6) | 2263 (57.1) |
|  | 2018 | 5572 | 2758 (49.5) | 33 (15.7) | 1143 (20.5) | 99 (16) | 1432 (25.7) | 455 (39.8) |
| **Burundi** | 2013 | 3750 | 1871 (49.9) | 32.8 (15.6) | 772 (20.6) | 112 (16) | 371 (9.9) | 268 (34.7) |
| **Cameroon** | 2019 | 4732 | 2340 (49.5) | 32.5 (15.5) | 1203 (25.4) | 106 (16) | 702 (14.8) | 429 (35.7) |
| **DRC^c^** | 2014 | 8216 | 4119 (50.1) | 32.5 (15.6) | 2958 (36) | 103 (17) | 1644 (20) | 1098 (37.1) |
| **Côte d’Ivoire** | 2012 | 3391 | 1710 (50.4) | 31.6 (15.4) | 1530 (45.1) | 99 (16) | 889 (26.2) | 568 (37.1) |
| **Gambia** | 2013 | 3139 | 1504 (47.9) | 31.4 (15.6) | 56 (1.8) | 98 (16) | 869 (27.7) | 24 (42.9) |
| **Ghana** | 2014 | 2722 | 1306 (48) | 32.7 (15.3) | 1112 (40.9) | 101 (15) | 584 (21.5) | 429 (38.6) |
|  | 2016 | 3078 | 1503 (48.8) | 32.3 (15.5) | 998 (32.4) | 102 (15) | 554 (18) | 349 (35) |
|  | 2019 | 2867 | 1403 (48.9) | 32.6 (15.6) | 718 (25) | 105 (14) | 384 (13.4) | 205 (28.6) |
| **Guinea** | 2012 | 3215 | 1533 (47.7) | 32.3 (15.3) | 1465 (45.6) | 97 (18) | 971 (30.2) | 687 (46.9) |
| **Kenya** | 2015 | 3426 | 1698 (49.6) | 33.3 (15.4) | 317 (9.3) | 116 (16) | 196 (5.7) | 56 (17.7) |
| **Liberia** | 2009 | 4049 | 2029 (50.1) | 32.5 (15.7) | 1443 (35.6) | 104 (14) | 595 (14.7) | 359 (24.9) |
|  | 2011 | 3184 | 1569 (49.3) | 33 (15.3) | 1635 (51.4) | 99 (15) | 802 (25.2) | 599 (36.6) |
|  | 2016 | 2787 | 1398 (50.2) | 33 (15.5) | 1409 (50.6) | 98 (15) | 760 (27.3) | 545 (38.7) |
| **Madagascar** | 2011 | 6235 | 3076 (49.3) | 33.7 (15.6) | 384 (6.2) | 109 (14) | 455 (7.3) | 75 (19.5) |
|  | 2013 | 5426 | 2669 (49.2) | 32.7 (15.5) | 399 (7.4) | 110 (15) | 484 (8.9) | 114 (28.6) |
|  | 2016 | 6931 | 3368 (48.6) | 32.5 (15.1) | 252 (3.6) | 111 (14) | 476 (6.9) | 58 (23) |
| **Malawi** | 2012 | 2115 | 1120 (53) | 32.3 (15.3) | 813 (38.4) | 103 (16) | 363 (17.2) | 275 (33.8) |
|  | 2014 | 1921 | 956 (49.8) | 32.4 (15.6) | 578 (30.1) | 109 (15) | 206 (10.7) | 132 (22.8) |
|  | 2017 | 2308 | 1153 (50) | 33.7 (16) | 603 (26.1) | 107 (15) | 308 (13.3) | 172 (28.5) |
| **Mali** | 2013 | 4697 | 2307 (49.1) | 33.3 (15.5) | 2070 (44.1) | 94 (18) | 1737 (37) | 1107 (53.5) |
|  | 2015 | 7299 | 3586 (49.1) | 32.7 (15.6) | 2292 (31.4) | 93 (17) | 2913 (39.9) | 1366 (59.6) |
|  | 2018 | 4339 | 2115 (48.7) | 32.5 (15.3) | 680 (15.7) | 96 (16) | 1363 (31.4) | 354 (52.1) |
| **Mozambique** | 2011 | 4915 | 2509 (51) | 31.8 (15.5) | 1668 (33.9) | 103 (16) | 933 (19) | 627 (37.6) |
|  | 2015 | 4434 | 2265 (51.1) | 32.4 (15.2) | 1400 (31.6) | 105 (17) | 720 (16.2) | 449 (32.1) |
|  | 2018 | 4362 | 2201 (50.5) | 32.2 (15.5) | 1500 (34.4) | 97 (17) | 1298 (29.8) | 742 (49.5) |
| **Nigeria** | 2010 | 4923 | 2432 (49.4) | 32.7 (15.8) | 2316 (47) | 99 (18) | 1256 (25.5) | 867 (37.4) |
|  | 2015 | 6021 | 2986 (49.6) | 32.9 (15.4) | 2624 (43.6) | 101 (16) | 1345 (22.3) | 976 (37.2) |
|  | 2018 | 11195 | 5535 (49.4) | 32.2 (15.6) | 4257 (38) | 101 (16) | 2391 (21.4) | 1656 (38.9) |
| **Rwanda** | 2008 | 4622 | 2306 (49.9) | 32.1 (15.6) | 94 (2) | 118 (14) | 150 (3.2) | 26 (27.7) |
|  | 2011 | 4019 | 1986 (49.4) | 33.4 (15.4) | 97 (2.4) | 119 (14) | 90 (2.2) | 13 (13.4) |
|  | 2015 | 3446 | 1689 (49) | 32 (15.3) | 259 (7.5) | 118 (14) | 130 (3.8) | 68 (26.3) |
| **Senegal** | 2009 | 4026 | 2003 (49.8) | 33.1 (15.2) | 482 (12) | 95 (18) | 1457 (36.2) | 288 (59.8) |
|  | 2011 | 3866 | 1843 (47.7) | 32.6 (15.6) | 126 (3.3) | 97 (17) | 1147 (29.7) | 66 (52.4) |
|  | 2013 | 5777 | 2870 (49.7) | 32.2 (15.4) | 234 (4.1) | 99 (17) | 1471 (25.5) | 138 (59) |
|  | 2014 | 6234 | 3081 (49.4) | 32.7 (15.4) | 111 (1.8) | 105 (16) | 985 (15.8) | 42 (37.8) |
|  | 2015 | 6239 | 3099 (49.7) | 32.8 (15.3) | 61 (1) | 101 (15) | 1335 (21.4) | 28 (45.9) |
|  | 2016 | 6077 | 2991 (49.2) | 33.1 (15.3) | 110 (1.8) | 102 (15) | 1142 (18.8) | 53 (48.2) |
|  | 2017 | 10894 | 5371 (49.3) | 32.6 (15.6) | 169 (1.6) | 100 (15) | 2387 (21.9) | 110 (65.1) |
| **Sierra Leone** | 2016 | 6659 | 3314 (49.8) | 32.3 (15.7) | 3785 (56.8) | 97 (16) | 1928 (29) | 1511 (39.9) |
| **Tanzania** | 2012 | 7690 | 3804 (49.5) | 32.1 (15.5) | 762 (9.9) | 108 (16) | 870 (11.3) | 197 (25.9) |
|  | 2016 | 9283 | 4656 (50.2) | 31.9 (15.6) | 1112 (12) | 108 (15) | 1029 (11.1) | 336 (30.2) |
| **Togo** | 2014 | 3218 | 1591 (49.4) | 32.5 (15.6) | 1260 (39.2) | 101 (15) | 718 (22.3) | 472 (37.5) |
|  | 2017 | 3202 | 1607 (50.2) | 32.3 (15.8) | 1519 (47.4) | 99 (15) | 800 (25) | 553 (36.4) |
| **Uganda** | 2010 | 3612 | 1828 (50.6) | 32.3 (15.5) | 2041 (56.5) | 104 (18) | 733 (20.3) | 622 (30.5) |
|  | 2015 | 4469 | 2292 (51.3) | 32.8 (15.3) | 1556 (34.8) | 109 (16) | 492 (11) | 329 (21.1) |
|  | 2016 | 4788 | 2378 (49.7) | 32.5 (15.4) | 1594 (33.3) | 109 (16) | 565 (11.8) | 384 (24.1) |
|  | 2019 | 6972 | 3435 (49.3) | 32.8 (15.6) | 1596 (22.9) | 110 (15) | 632 (9.1) | 332 (20.8) |

**S6. Subset of twins.** Describes demographic characteristics for twins within each included survey. ^a^Prevalence of moderate to severe anemia among the twins (Hb < 90 g/L). ^b^Prevalence of moderate to severe anemia (< 90 g/L) among malaria positive twins. ^c^Democratic Republic of Congo.

| **Survey** | | **Twins** | **Female** | **Age (months)** | **Malaria Positive** | **Hb [g/L]** | **Pv Anemia^a^** | **Pv Anemia^b^** |
| --- | --- | --- | --- | --- | --- | --- | --- | --- |
| **Country** | **Year** | **N** | **N (%)** | **Mean (SD)** | **N (%)** | **Mean (SD)** | **N (%)** | **N (%)** |
| **Angola** | 2007 | 62 | 27 (43.5) | 31.3 (18.8) | 19 (30.6) | 108 (14) | 7 (11.3) | 5 (26.3) |
|  | 2011 | 126 | 58 (46) | 38.5 (16.6) | 24 (19) | 109 (14) | 9 (7.1) | 5 (20.8) |
|  | 2016 | 167 | 90 (53.9) | 31.7 (15.6) | 29 (17.4) | 102 (14) | 29 (17.4) | 12 (41.4) |
| **Benin** | 2012 | 137 | 73 (53.3) | 34.3 (16.1) | 29 (21.2) | 106 (17) | 20 (14.6) | 7 (24.1) |
|  | 2018 | 226 | 114 (50.4) | 29 (15.9) | 71 (31.4) | 99 (17) | 50 (22.1) | 27 (38) |
| **Burkina Faso** | 2010 | 136 | 60 (44.1) | 30.4 (15) | 106 (77.9) | 88 (17) | 73 (53.7) | 57 (53.8) |
|  | 2014 | 168 | 88 (52.4) | 28.3 (14.7) | 105 (62.5) | 88 (16) | 92 (54.8) | 61 (58.1) |
|  | 2018 | 171 | 93 (54.4) | 31 (15.4) | 28 (16.4) | 99 (17) | 49 (28.7) | 10 (35.7) |
| **Burundi** | 2012 | 65 | 29 (44.6) | 26.6 (17.3) | 9 (13.8) | 109 (19) | 11 (16.9) | 4 (44.4) |
| **Cameroon** | 2018 | 185 | 99 (53.5) | 34.1 (16.5) | 50 (27) | 106 (16) | 29 (15.7) | 16 (32) |
| **DRC^c^** | 2014 | 202 | 97 (48) | 32.3 (15) | 80 (39.6) | 100 (17) | 56 (27.7) | 37 (46.2) |
| **Côte d’Ivoire** | 2012 | 104 | 55 (52.9) | 28.2 (14.7) | 52 (50) | 94 (16) | 38 (36.5) | 24 (46.2) |
| **Gambia** | 2013 | 69 | 35 (50.7) | 29.1 (14.6) | 1 (1.4) | 95 (19) | 30 (43.5) | 1 (100) |
| **Ghana** | 2014 | 120 | 62 (51.7) | 31.9 (15.9) | 47 (39.2) | 102 (16) | 26 (21.7) | 15 (31.9) |
|  | 2016 | 116 | 70 (60.3) | 30.9 (14.6) | 40 (34.5) | 99 (15) | 29 (25) | 19 (47.5) |
|  | 2019 | 88 | 43 (48.9) | 35 (14.6) | 10 (11.4) | 104 (15) | 14 (15.9) | 2 (20) |
| **Guinea** | 2012 | 86 | 46 (53.5) | 34.6 (14.6) | 46 (53.5) | 96 (17) | 29 (33.7) | 20 (43.5) |
| **Kenya** | 2015 | 82 | 44 (53.7) | 37 (14.8) | 15 (18.3) | 114 (19) | 10 (12.2) | 2 (13.3) |
| **Liberia** | 2009 | 113 | 54 (47.8) | 34.8 (15.3) | 35 (31) | 104 (14) | 19 (16.8) | 5 (14.3) |
|  | 2011 | 94 | 43 (45.7) | 35.3 (15.2) | 52 (55.3) | 98 (15) | 26 (27.7) | 21 (40.4) |
|  | 2016 | 74 | 33 (44.6) | 30.4 (16.3) | 44 (59.5) | 96 (15) | 29 (39.2) | 21 (47.7) |
| **Madagascar** | 2011 | 129 | 75 (58.1) | 37.2 (14.9) | 10 (7.8) | 108 (16) | 13 (10.1) | 2 (20) |
|  | 2013 | 82 | 41 (50) | 37.2 (16.3) | 12 (14.6) | 109 (17) | 10 (12.2) | 4 (33.3) |
|  | 2016 | 117 | 50 (42.7) | 31.6 (13.8) | 1 (0.9) | 108 (16) | 17 (14.5) | 1 (100) |
| **Malawi** | 2012 | 78 | 38 (48.7) | 33.6 (14.6) | 37 (47.4) | 106 (15) | 10 (12.8) | 6 (16.2) |
|  | 2014 | 66 | 36 (54.5) | 29.4 (18.5) | 16 (24.2) | 106 (21) | 17 (25.8) | 5 (31.2) |
|  | 2017 | 46 | 27 (58.7) | 25.7 (15.3) | 11 (23.9) | 104 (17) | 9 (19.6) | 2 (18.2) |
| **Mali** | 2013 | 125 | 65 (52) | 33 (15.6) | 48 (38.4) | 95 (21) | 42 (33.6) | 24 (50) |
|  | 2015 | 200 | 105 (52.5) | 34 (16.1) | 56 (28) | 92 (17) | 80 (40) | 33 (58.9) |
|  | 2018 | 124 | 60 (48.4) | 33.1 (14.6) | 14 (11.3) | 96 (16) | 44 (35.5) | 8 (57.1) |
| **Mozambique** | 2011 | 142 | 71 (50) | 32.6 (14.1) | 45 (31.7) | 104 (14) | 21 (14.8) | 12 (26.7) |
|  | 2015 | 118 | 62 (52.5) | 31.6 (14.9) | 28 (23.7) | 106 (14) | 12 (10.2) | 5 (17.9) |
|  | 2018 | 90 | 47 (52.2) | 30.1 (15.4) | 32 (35.6) | 98 (16) | 25 (27.8) | 12 (37.5) |
| **Nigeria** | 2010 | 132 | 62 (47) | 32.4 (15.5) | 52 (39.4) | 104 (15) | 21 (15.9) | 12 (23.1) |
|  | 2015 | 188 | 101 (53.7) | 33.9 (16.4) | 79 (42) | 102 (17) | 41 (21.8) | 31 (39.2) |
|  | 2018 | 282 | 149 (52.8) | 34.2 (16.9) | 92 (32.6) | 102 (15) | 58 (20.6) | 31 (33.7) |
| **Rwanda** | 2008 | 81 | 50 (61.7) | 30.6 (15.1) | 2 (2.5) | 113 (15) | 5 (6.2) | 0 (0) |
|  | 2011 | 76 | 40 (52.6) | 33.9 (15) | 1 (1.3) | 118 (15) | 2 (2.6) | 0 (0) |
|  | 2015 | 54 | 27 (50) | 34.3 (14.6) | 3 (5.6) | 117 (18) | 4 (7.4) | 1 (33.3) |
| **Senegal** | 2009 | 98 | 50 (51) | 31.6 (15) | 11 (11.2) | 88 (2) | 51 (52) | 7 (63.6) |
|  | 2011 | 123 | 61 (49.6) | 31.4 (16.1) | 6 (4.9) | 95 (16) | 42 (34.1) | 2 (33.3) |
|  | 2013 | 176 | 91 (51.7) | 28.7 (14.3) | 7 (4) | 94 (19) | 70 (39.8) | 6 (85.7) |
|  | 2014 | 179 | 95 (53.1) | 29.7 (15.3) | 8 (4.5) | 101 (19) | 47 (26.3) | 3 (37.5) |
|  | 2015 | 169 | 83 (49.1) | 31.4 (15.9) | 1 (0.6) | 101 (15) | 34 (20.1) | 1 (100) |
|  | 2016 | 185 | 84 (45.4) | 30.9 (14.4) | 4 (2.2) | 98 (15) | 44 (23.8) | 2 (50) |
|  | 2017 | 292 | 160 (54.8) | 32.8 (15) | 5 (1.7) | 98 (17) | 85 (29.1) | 4 (80) |
| **Sierra Leone** | 2016 | 244 | 138 (56.6) | 29.6 (15.4) | 132 (54.1) | 93 (17) | 93 (38.1) | 62 (47) |
| **Tanzania** | 2012 | 200 | 98 (49) | 28.5 (15.5) | 20 (10) | 101 (17) | 53 (26.5) | 6 (30) |
|  | 2016 | 265 | 152 (57.4) | 32.8 (15.4) | 25 (9.4) | 105 (17) | 43 (16.2) | 10 (40) |
| **Togo** | 2014 | 94 | 46 (48.9) | 29.2 (17.5) | 38 (40.4) | 98 (17) | 29 (30.9) | 12 (31.6) |
|  | 2017 | 120 | 62 (51.7) | 31.8 (16.5) | 56 (46.7) | 92 (18) | 55 (45.8) | 36 (64.3) |
| **Uganda** | 2010 | 104 | 53 (51) | 34.7 (15.8) | 70 (67.3) | 102 (17) | 20 (19.2) | 12 (17.1) |
|  | 2015 | 110 | 55 (50) | 28.9 (15.4) | 40 (36.4) | 106 (18) | 21 (19.1) | 9 (22.5) |
|  | 2016 | 138 | 61 (44.2) | 29.1 (16) | 45 (32.6) | 103 (18) | 27 (19.6) | 13 (28.9) |
|  | 2019 | 166 | 82 (49.4) | 34.6 (15.8) | 40 (24.1) | 109 (16) | 17 (10.2) | 11 (27.5) |

**S7.** **Hemoglobin distribution among twins in the analysis population by malaria status**. Mean (SD) hemoglobin for malaria negative children is 103g/L (17 g/L), the median is 104g/L. Mean (SD) hemoglobin for malaria positive children is 94 g/L (18g/L), the median is 94g/L.

***
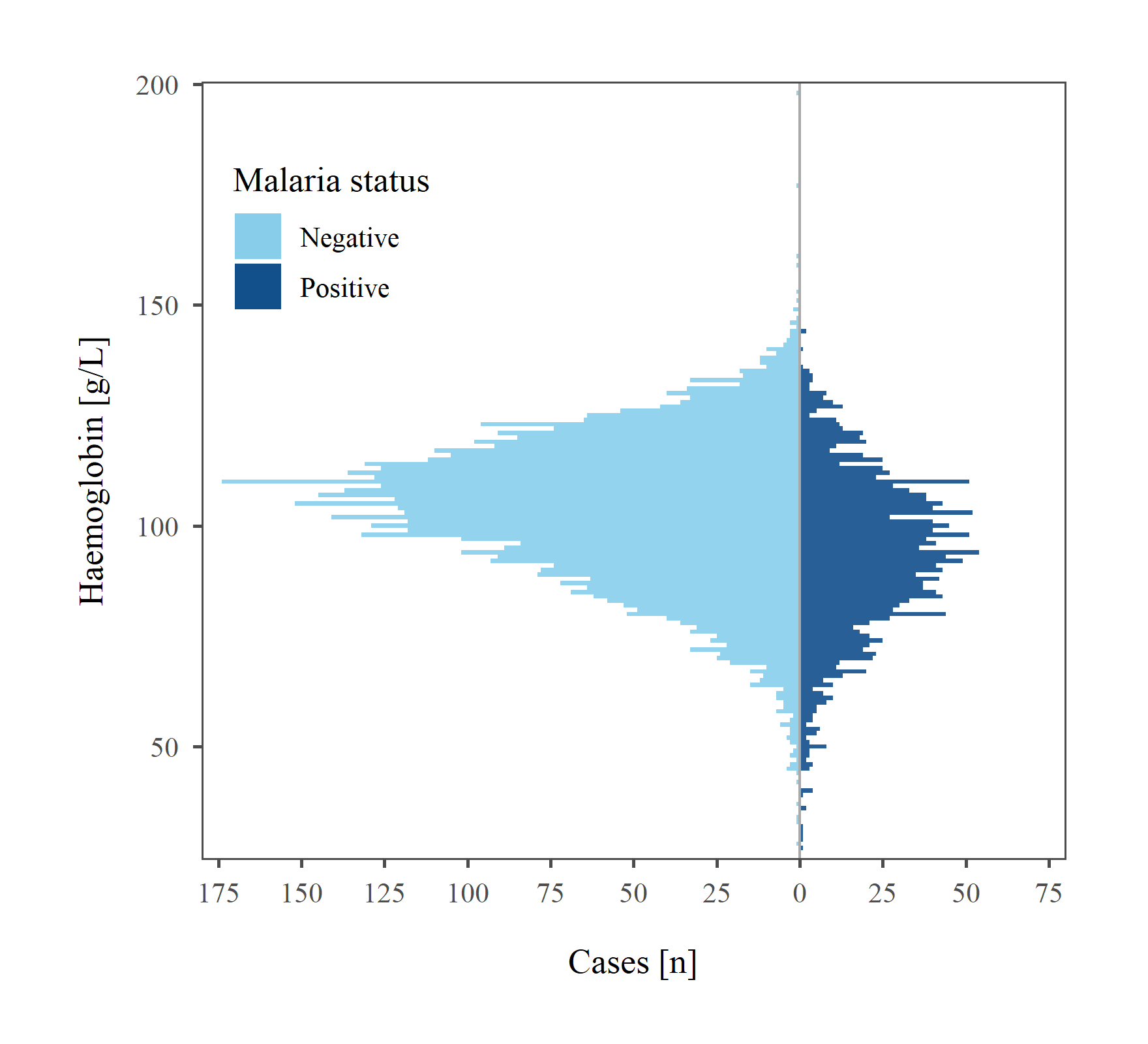
***

**S8. Regression results for the subset analysis by age.** The bars represent the regression model outcome as change from the model main intercept (Baseline Hb) with 95% Confidence Intervals. Age appears to have a substantial modulatory impact on the malaria effect on hemoglobin. The malaria effect is strongest among the youngest children and decreases until the age group of 25 – 36 month olds. After this, the trend briefly reverses, increasing in the 37 – 48 year olds and finally decreasing again in the 49 – 59 year olds. The horizontal line is the line of no effect.


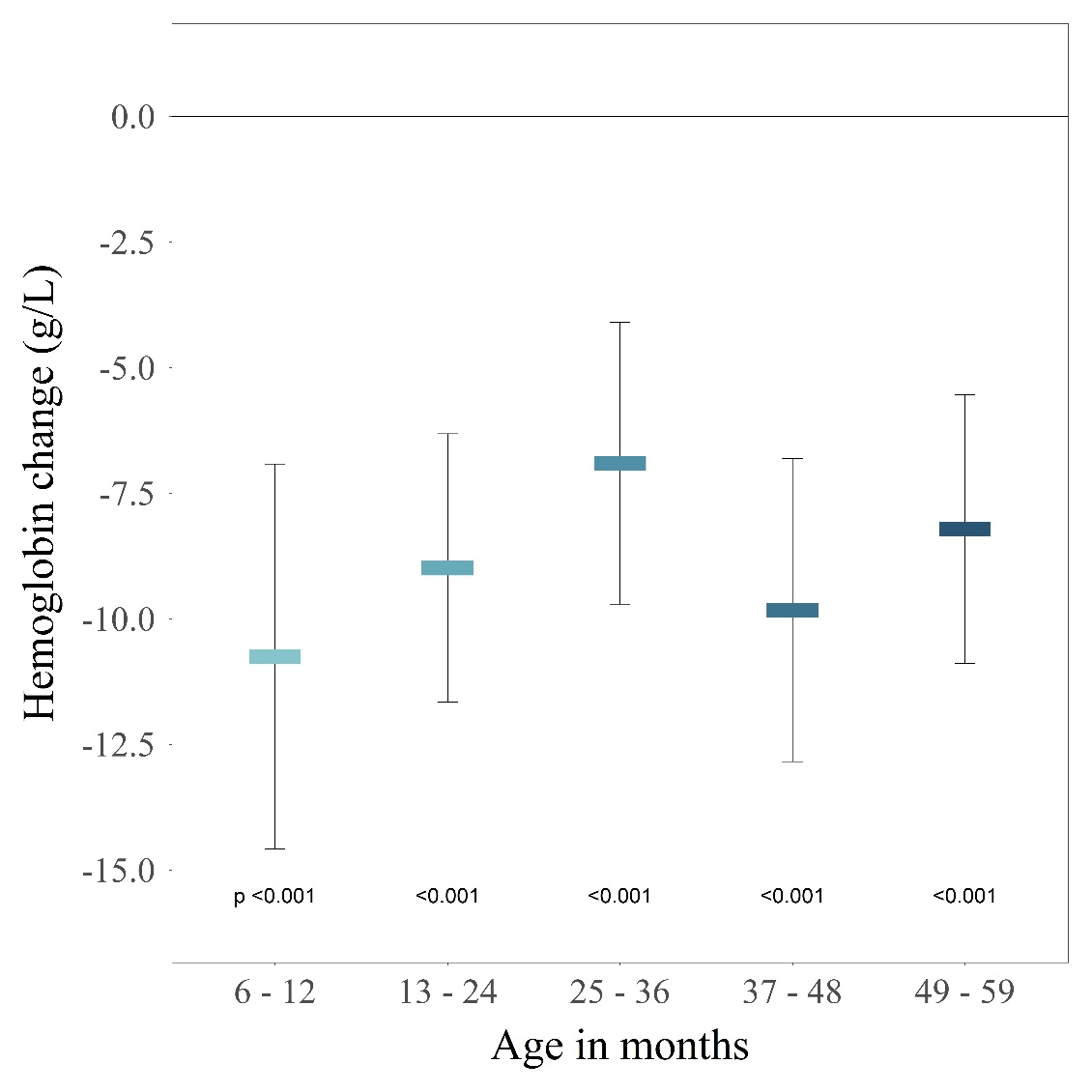


**S9. The malaria effect on hemoglobin among twins in Sub-saharan Africa.** Regression results of the main model indicate the malaria induced hemoglobin change. The table presents the outcomes for the main model of all pooled twins, the same-sex subset analysis and different-sex subset analysis. We adjusted for sex, where applicable. Hemoglobin is given in g/L units.

| **Coefficients** |  | **Model** | | |
| --- | --- | --- | --- | --- |
|  |  | **Main** | **Same-Sex** | **Different-Sex** |
| **Malaria Status** |  |  |  |  |
| Reference: Negative | |  |  |  |
| Positive | Estimate^a^ | -9 | -9 | -8 |
|  | p-value | < 0.001 | < 0.001 | < 0.001 |
|  | 95% CI | (-10; -7) | (-11; -7) | (-10; -6) |
| **Sex** |  |  |  |  |
| Reference: Male | |  |  |  |
| Female | Estimate^a^ | 2 |  | 2 |
|  | p-value | < 0.001 |  | < 0.001 |
|  | 95% CI | (1; 3) |  | (1; 3) |

^a^ marks the change [g/L] in hemoglobin in the presence of *plasmodium spp*

**S10. Quasi-Poisson regression results of the malaria effect on the relative risk of anemia by severity.** Malaria positive children had a higher relative risk of mild or worse anemia (Hb < 110 g/L; RR 1.28, p < 0.001, CI95% 1.2; 1.36), moderate or worse anemia (Hb < 90 g/L, RR 1.76, p < 0.001, CI95% 1.49; 2.08) and severe anemia (Hb < 70 g/L, RR 3.01, p < 0.001, CI95% 1.79; 5.1). At the same time, their “risk” of being healthy was greatly reduced (Hb ≥ 110; RR 0.51, p < 0.001, CI95% 0.43; 0.61). The error bars represent the 95% confidence intervals of each analysis. The horizontal line is the line of no effect.


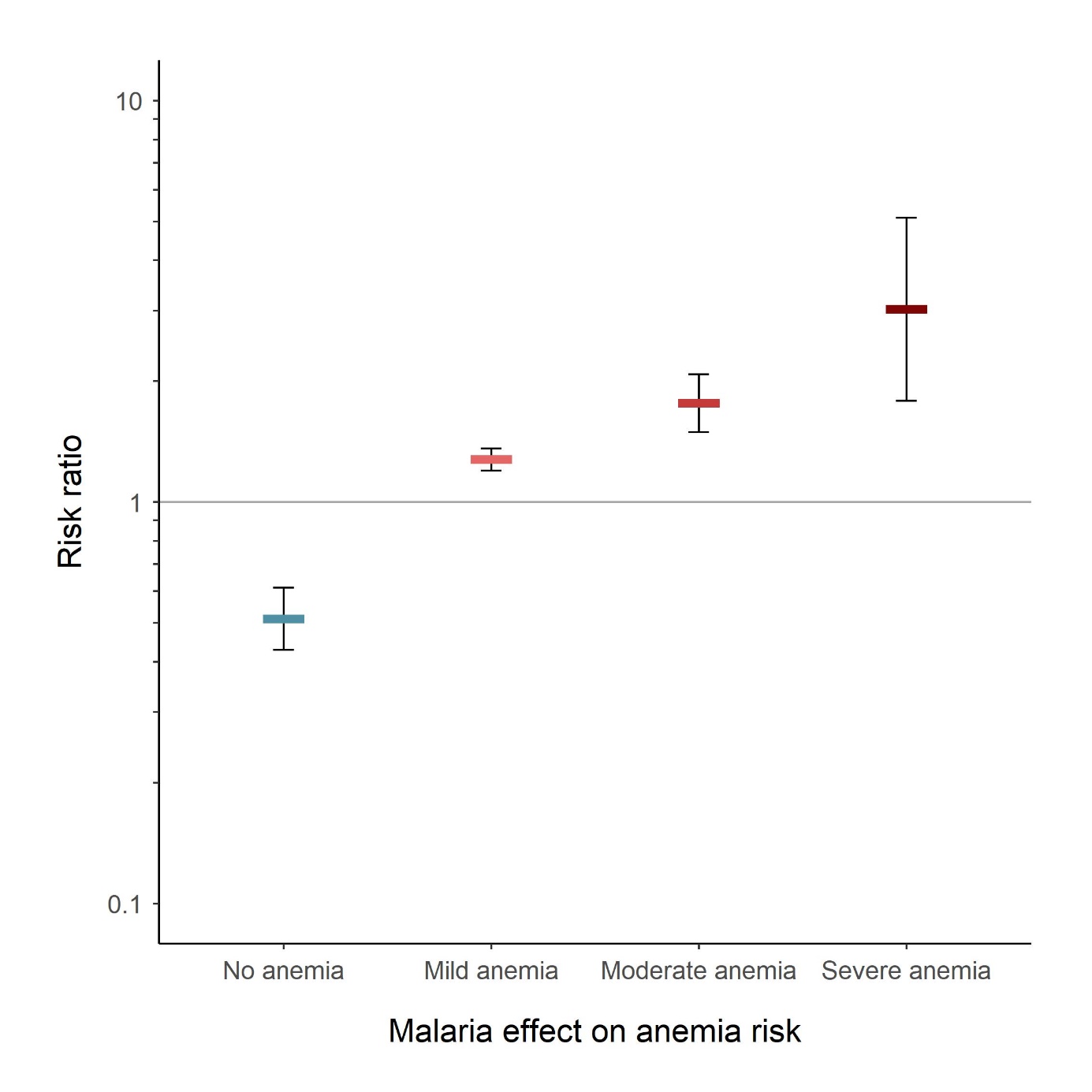


**References**

1. Fellman J, Eriksson AW. Weinberg's differential rule reconsidered. *Hum Biol*. Jun 2006;78(3):253-75. doi:10.1353/hub.2006.0044

2. Price RN, Simpson JA, Nosten F, et al. Factors contributing to anemia after uncomplicated falciparum malaria. *Am J Trop Med Hyg*. 01 Nov. 2001 2001;65(5):614-622. doi:10.4269/ajtmh.2001.65.614

3. Douglas NM, Lampah DA, Kenangalem E, et al. Major Burden of Severe Anemia from Non-Falciparum Malaria Species in Southern Papua: A Hospital-Based Surveillance Study. *PLOS Medicine*. 2013;10(12):e1001575. doi:10.1371/journal.pmed.1001575

4. Jaenisch T, Sazawal S, Dutta A, Deb S, Ramsan M, Sullivan DJ. Contributions of polyclonal malaria, gametocytemia, and pneumonia to infant severe anemia incidence in malaria hyperendemic Pemba, Tanzania. *Am J Trop Med Hyg*. Jun 2012;86(6):925-30. doi:10.4269/ajtmh.2012.11-0164
